# Molecular Simulation of Coal Molecular Diffusion Properties in Chicheng Coal Mine

**DOI:** 10.3390/molecules28196933

**Published:** 2023-10-04

**Authors:** Jingxue Yan, Baoshan Jia, Baogang Liu, Jinyi Zhang

**Affiliations:** 1 College of Safety Science and Engineering, Liaoning Technical University, Fuxin 123000, China; yanjingxue0308@163.com (J.Y.); m18941829785@163.com (B.L.); zjylntu@163.com (J.Z.); 2 Laboratory of Mine Thermal Power Disaster and Prevention, Ministry of Education, Liaoning Technical University, Fuxin 123000, China

**Keywords:** coal bed methane, molecular simulation, diffusion characteristics, micro-mechanism, coal seam extraction

## Abstract

In order to study the importance of the diffusion mechanism of CH_4_ and CO_2_ in coal for the development of coalbed methane, the aim of this paper is to reveal the influence mechanism of pressure, temperature, water content and other factors on the molecular diffusion behavior of gas at the molecular level. In this paper, non-sticky coal in Chicheng Coal Mine is taken as the research object. Based on the molecular dynamics method (MD) and Monte Carlo (GCMC) method, the diffusion characteristics and microscopic mechanism of CH_4_ and CO_2_ in coal under different pressures (100 kPa–10 MPa), temperatures (293.15–313.15 K) and water contents (1–5%) were analyzed in order to lay a theoretical foundation for revealing the diffusion characteristics of CBM in coal, and provide technical support for further improving CBM extraction. The results show that high temperature is conducive to gas diffusion, while high pressure and water are not conducive to gas diffusion in the coal macromolecular model.

## 1. Introduction

The gas diffusion performance in coal is an important parameter that affects the gas production rate of coalbed methane wells and determines the final production of coalbed methane [1]. Gas diffusion in coal is due to the slow movement of gas from high concentration to low concentration under the condition of concentration difference, and it presents a process of uniform distribution after a long time [2]. Gas diffusion is an inefficient means of material transport. Diffusion coefficient is commonly used to characterize the diffusion degree of gas in coal seam. The diffusion coefficient of gas in coal can be obtained via the molecular dynamics simulation method. So far, many scholars have studied the diffusion properties of CH_4_ and CO_2_ gases in coal. Diffusion is an important part of gas migration in coal, which is usually related to gas type, moisture, gas pressure and temperature [3].

Li Bin [4], taking anthracite, coking coal and long-flame coal as research objects, carried out experiments on adsorption–deformation–seepage of CO_2_, CH_4_ and CH_4_ in coal of different rank under stress, and studied molecular simulation of adsorption and diffusion behavior of CO_2_, CH_4_ and CH_4_ in different coal macromolecular models. Miao Zhang et al. [5] carried out an adsorption–desorption diffusion test of CO_2_ in coal particles under different temperature and pressure conditions, and used different adsorption and diffusion models to fit and analyze the test results. Dai Xuanyan [6] studied the adsorption and diffusion states of single and mixed components of (1:1) CH_4_ and CO_2_ of three minerals (illite, montmorillonite and calcite), and found that the self-diffusion coefficients of CH_4_ and CO_2_ first decreased and then increased with an increase in buried depth. Junlin Liu et al. [7] studied the diffusion behavior of CO_2_ and CH_4_ gases in the CO_2_-ECBM process by taking the pore characteristics of coal reservoirs of 13 coals in Liuzhuang Mine and 7 coals in Qidong Mine in the low-permeability coal-bearing area of Lianghuai, China, as the research object. Kaiyuan Li [8], based on coal gangue samples of different particle-size groups as research objects, simulated the characteristics of CO_2_ diffusion over time in porous media samples by using laboratory isothermal adsorption experiments, equation fitting, software simulation and other methods. You et al. [9] used molecular simulation to replace a lignite model with graphite surface containing OH, -COOH and carbonyl groups. By analyzing the radial distribution function and diffusion coefficient of H_2_O molecules, it was concluded that -COOH was the preferred adsorption site. Hu et al. [10] compared the diffusion characteristics of CO_2_ and CH_4_ in coal, and found that the CO_2_ diffusion coefficient was about 10^−9^ m^2^/s. An Fenghua [11] studied the diffusion coefficient under different stress, concentration gradient, temperature and gas type conditions with the direct steady-state method based on Fick’s law, and the results show that the diffusion coefficient of gas has a negative linear relationship with stress. Xu et al. [12] proposed a new laboratory measurement method for methane diffusion coefficient in coal matrix, using coal matrix flakes instead of coal particles as measurement samples. By means of molecular simulation, Yu Song [13] et al. studied the diffusion characteristics of CO_2_ and CH_4_ molecules using a Wise bituminous coal macromolecular structure model, indicating that CO_2_ and CH_4_ are mainly diffused via micropores in the coal model. Liu et al. [14] studied the effect of coal type size on gas diffusion of pulverized coal and lump coal under unconstrained conditions, and the results showed that there was a scale effect on gas diffusion in coal. Keshavarz et al. [15] studied the effects of maceral composition and coal rank on the diffusion rate of CO_2_ and CH_4_ in 18 Australian bituminous and subbituminous coals. Hu et al. [16] further established a simplified numerical method for a dual-dispersion diffusion model and compared it with the experimental results. Hu et al. [17] studied the self-diffusion and mutual diffusion of CO_2_-CH_4_ mixture via molecular simulation, and the results showed that the self-diffusion coefficient decreased with an increase in gas concentration and increased with an increase in temperature.

In the article “Simulation study on molecular adsorption of coal in Chicheng Coal Mine”, I studied the adsorption characteristics of coal and obtained the following results: In the macromolecular structure model of dry coal, under the same conditions, the adsorption capacity, interaction energy and adsorption heat of CO_2_ were all greater than that of CH_4_, and CO_2_ was more sensitive to temperature changes. The equivalent adsorption heat of CO_2_ and CH_4_ adsorbed in wet coal with different water content decreased with an increase in pressure and increased with an increase in water content [18]. The diffusion mechanism of CH_4_ and CO_2_ in coal is analyzed in this paper. The effects of temperature, pressure and water content on the diffusion of CO_2_ and CH_4_ on the macromolecular structure of non-stick coal are mainly studied, which is very important for the development of coalbed methane.

## 2. Results and Discussion

### 2.1. Structural Characterization and Construction of Macromolecular Structure of Coal

This study selected the non-caking coal of Chicheng Coal Mine as the research object, and industrial/elemental analysis, such as FTIR, XPS and solid [15] C NMR (Ceshigou Research Service, Beijing, China), was used to characterize and analyze the organic matter in the coal sample, including the aromatic structures, oxygen-containing functional groups, fatty carton structure, occurrence state of sulfur element and other parameter characteristics, on the basis of which a coal macromolecular structure model was constructed. This analysis provided support for the construction ideas and methods of the coal macromolecular structure model [18].

**Figure 1:**
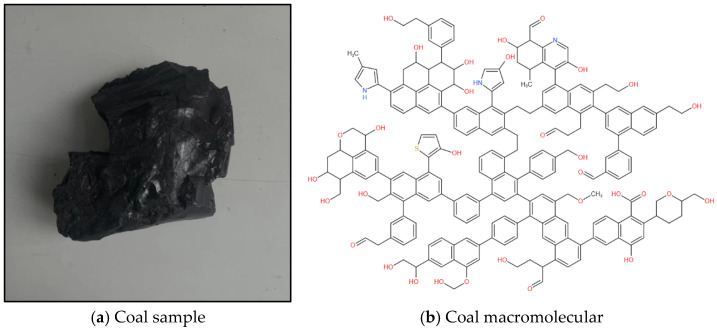
Test coal sample and plane model of coal macromolecular structure.

The study of the physicochemical structure of coal can enable a complete understanding of the adsorption performance of coal for gas [19]. In this study, fresh coal samples (density 1.16 g/cm^3^, *R*°_max_ 0.665%) from the 1502−2 working face of Chicheng Coal Mine were selected. The coal samples were crushed, screened and divided using a crusher and a vibrating screen machine to produce analytical samples with a particle size below 200 mesh. Based on the results of the elemental analysis, Fourier-transform infrared spectroscopy (FT−IR), X-ray photoelectron spectroscopy (XPS) and carbon-13 nuclear magnetic resonance (^13^C NMR) experimental characterization, the molecular formula of non-sticky coal in Chicheng Coal Mine was determined to be C_207_H_181_O_32_N_3_S (C: 76.39%, N: 1.29%, O: 15.73%, H: 5.61%, S: 0.99). The coal macromolecular model is shown in Figure 1 [18].

**Figure 2:**
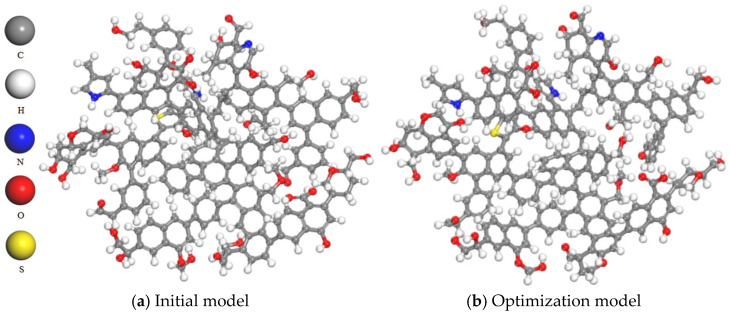
Comparison before and after model optimization.

The two-dimensional plane model of coal macromolecules shown in Figure 1 was imported into the MS molecular simulation software to construct an initial three-dimensional structure, as shown in Figure 2a. The Forcite module for geometric optimization of the model was used and the COMPASS force field was selected for geometric optimization. The selection of the COMPASS force field is justified by its ability to provide a unified approach for modeling both organic and inorganic molecular systems. This force field can be applied to various types of molecules, including organics, polymers, gases and inorganics, utilizing a classified treatment approach that employs different models for different systems. Furthermore, it allows an accurate description even when mixing these two types of systems together. In comparison to the commonly used Dreiding force field, the COMPASS force field yields more precise results in terms of structure and binding energy calculations. The parameters are derived from ab initio parameterization and empirical optimization [20]. The forcefield was set to charge, the calculation accuracy was set to Fine, and the iteration step was set to 5000. The model was then subjected to annealing and the NVT ensemble was selected. The temperature of Nose was set to 300–600 K, and the number of cycles was set to five. Pneumatic parameters were specified accordingly. The model structure after dynamic optimization is shown in Figure 2b [18].

**Figure 3:**
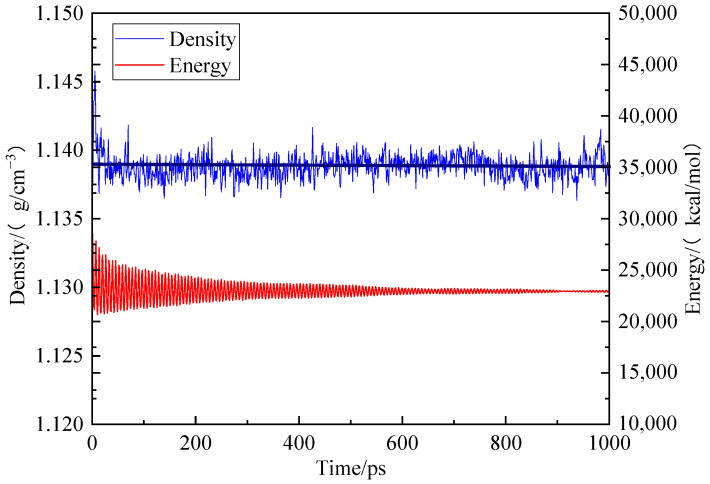
Trends of energy and concentration of different coal samples during kinetic optimization.

To establish the periodic boundary condition, the Amorphous Cell module was employed to put 10 optimized coal molecular models into the periodic cell. Firstly, the geometric optimization was conducted with predefined mechanical parameters. Subsequently, the model underwent annealing through a series of NPT cycles at temperatures ranging from 300 K to 600 K. A total of five cycles using the COMPASS force field, atom-based method for van der Waals term and Ewald method for electrostatic action term was performed. Finally, dynamic optimization was applied to the model with unchanged mechanical parameters as before. After kinetic treatment for 1000 ps, the total energy of the coal crystal cell model decreased and stabilized at its lowest value of 22,985.040 kcal/mol while maintaining a density of 1.138 g/cm^3^, which closely approximates that of real coal, as shown in Figure 3 [18].

**Figure 4:**
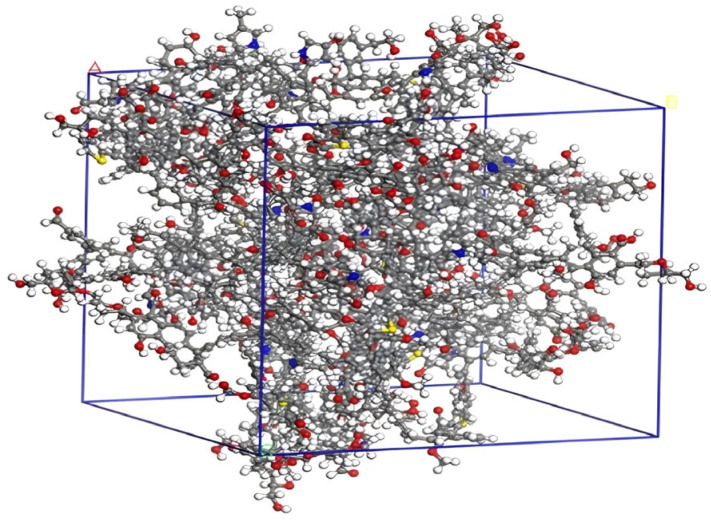
Coal macromolecular structure cell model.

The optimized coal macromolecular structure model is shown in Figure 4 (the structural model size of coal is *A* = *B* = *C* = 3.94357 nm), whose molecular formula is C_2070_H_1810_N_30_O_320_S_10_ [18].

### 2.2. Theoretical Formula of Gas Diffusion Characteristics of Coal

The Focite module was used to calculate the molecular dynamics of gas diffusion, and the mean azimuth shift curve of CO_2_ and CH_4_ gases in the non-stick coal large molecular structure was obtained. The root mean square shift (MSD) formula is as follows [21]:

(1) $$\begin{array}{l} MSD={\left|r_{i}\left(t\right)-r_{i}\left(0\right)\right|}^{2}\\ =\frac{1}{NN_{t}}\sum_{i=1}^{N}\sum_{t_{0}}^{N_{t}}\left|r_{i}\left(t+t_{0}\right)-r_{i}\left(t_{0}\right)\right|\\ =\underset{t\to \infty }{\lim}\left\{\frac{1}{N_{t}}\sum_{i=1}^{N}{\left[r_{i}\left(t\right)-r_{i}\left(0\right)\right]}^{2}\right\} \end{array}$$

where *r_i_*(*t*) and *r_i_*(0) are the position vectors at t time and initial time of the *i*-th gas molecule, respectively, in ps; *N_t_* is the number of molecular dynamics steps; and *t*_0_ is the initial time.

The diffusion coefficient of gas molecules in a coal macromolecular model can be obtained via the root-mean-square displacement curve and Einstein method, in which the formula of the Einstein method is as follows [22]:

(2) $$D=\frac{1}{6N}\lim\frac{d}{dt}{\left\{\sum_{i=1}^{N}\left[r_{i}\left(t\right)-r_{i}\left(0\right)\right]\right\}}^{2}$$

where *D* is the gas diffusion coefficient, in m^2^/s.

Through linear fitting of the gas mean azimuth shift curve in the coal molecular model, the slope can be obtained as $k^{\prime }$, as shown in Equation (3), and the diffusion coefficient can be simplified as shown in Equation (4) [21]:

(3) $$k^{\prime }=\underset{t\to \infty }{\lim}\frac{1}{t}\left\{\frac{1}{N_{t}}\sum_{i=1}^{N}{\left|r_{i}\left(t\right)-r_{i}\left(0\right)\right|}^{2}\right\}$$

(4) $$D=\frac{k^{\prime }}{6}$$

## 3. Materials and Methods

### 3.1. Influence of Temperature on Diffusion Performance

**Figure 5:**
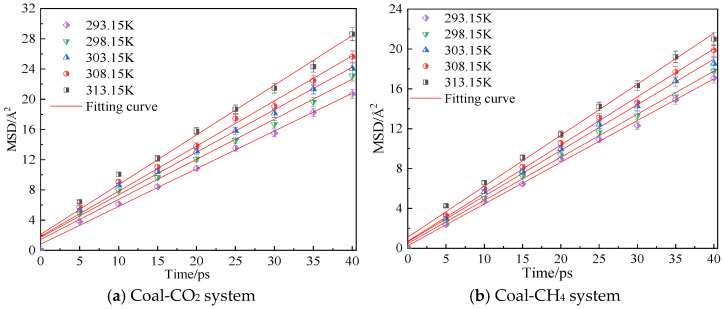
Mean square displacement curve of CO_2_/CH_4_ at different temperatures.

Figure 5 shows the mean azimuth shift curves of CO_2_ and CH_4_ at an adsorption pressure of 5 MPa and temperatures of 293.15 K, 298.15 K, 303.15 K, 308.15 K and 313.15 K. Through linear fitting of the mean azimuth shift curve, the diffusion coefficients of CO_2_ and CH_4_ at different temperatures can be obtained, as shown in Table 1. The simulation results show that the diffusion coefficients of CO_2_ and CH_4_ in the coal samples gradually increase with an increase in temperature. Under the same conditions, the diffusion coefficient of CH_4_ is smaller than that of CO_2_. The reason is that the kinetic energy of gas molecules increases with an increase in temperature, so the movement rate in the pores of coal body increases, which is conducive to the diffusion of gas molecules. The resistance to diffusion is smaller, so a high temperature can promote the diffusion rate of gas in coal.

To study the effect of temperature on gas diffusion, the diffusion activation energy of gas was calculated according to the Arrhenius equation, which is expressed as follows [22]:

(5) $$D=D_{0}e^{-\frac{E_{D}}{RT}}$$

where *D*_0_ refers to the pre-factor, in m^2^/s, and *E_D_* is the diffusion activation energy, in kJ/mol.

**Figure 6:**
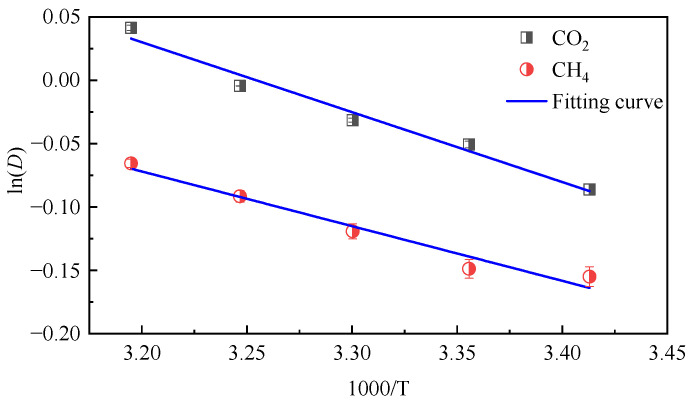
ln*D* of CO_2_/CH_4_ gas in coal molecules as a function of 1/T.

Using the diffusion coefficient calculated above, logarithm was taken on both sides of Equation (5), ln*D* and 1000/*T* curves were drawn, and the value of diffusion activation energy could be calculated through fitting. The fitting graph is shown in Figure 6.

The fitting results show that the diffusion activation energy of CO_2_ is 4.57 kJ·mol^−1^ and that of CH_4_ is 3.56 kJ·mol^−1^, and the activation energy of CO_2_ is greater than that of CH_4_ because the molecular diameter of CO_2_ is smaller than that of CH_4_, which is more favorable for diffusion in micropores. It also shows that the system of CO_2_ and coal molecules is more dependent on temperature.

**Figure 7:**
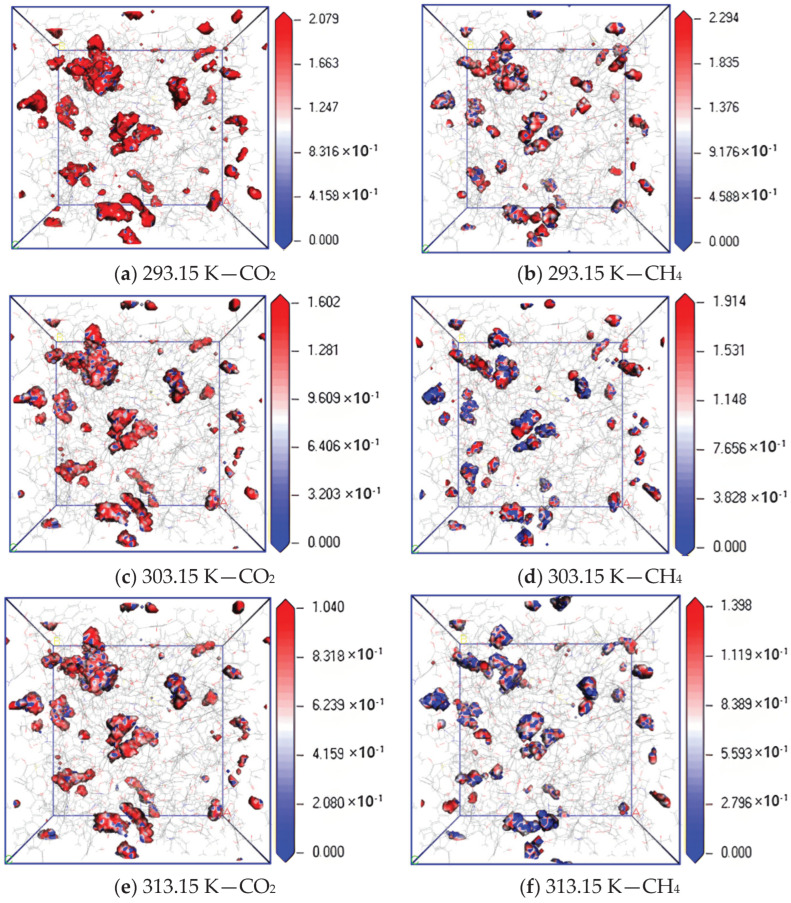
Isopotential value distribution of CO_2_ and CH_4_ at different temperatures.

To further study the diffusion mechanism of CO_2_ and CH_4_ in coal, the equipotential surface diagram of CO_2_ and CH_4_ at different temperatures with pressure of 5 MPa was obtained according to the trajectory file obtained via simulation calculation, as shown in Figure 7. The absolute value of equipotential value reflects the density of molecular distribution, and a lower equipotential value indicates a wider probability distribution, that is, the diffusion effect is better [22,23].

The simulation results show that when the adsorption temperature is 293.15 K, 303.15 K and 313.15 K, the maximum equipotential value of CO_2_ gas is 2.079, 1.602 and 1.040, and that of CH_4_ is 2.294, 1.914 and 1.398, respectively. The equipotential value of CH_4_ is greater than that of CO_2_. The maximum equipotential values of CO_2_ and CH_4_ gases in coal gradually decrease with an increase in temperature, which indicates that the two gases can be more widely and evenly distributed in the pores on the surface of coal molecules. The increase in temperature increases the movement frequency of nuclei and electrons inside gas molecules, which leads to the acceleration of gas diffusion rate in coal.

### 3.2. Influence of Pressure on Diffusion Performance

**Figure 8:**
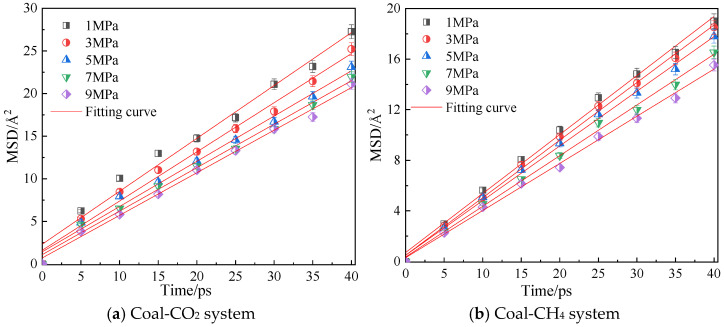
Mean square displacement curve of CO_2_/CH_4_ at different pressure.

Figure 8 shows the mean azimuth shift curves of CO_2_ and CH_4_ when the adsorption temperature is 298.15 K and the adsorption pressure is 1 MPa, 3 MPa, 5 MPa, 7 MPa and 9 MPa. Through linear fitting of the mean azimuth shift curve, the diffusion coefficients of CO_2_ and CH_4_ under different pressures are shown in Table 2. The simulation results show that the diffusion coefficients of CO_2_ and CH_4_ gradually decrease with an increase in pressure, indicating that a high pressure is not conducive to the diffusion of gas in coal, because with an increase in pressure, the average free path of the two gas molecules decreases [22], and they are more likely to collide with the surface of coal, thus inhibiting the diffusion of gas in coal. The diffusion coefficient of CO_2_ is always greater than that of CH_4_ under the same pressure.

**Figure 9:**
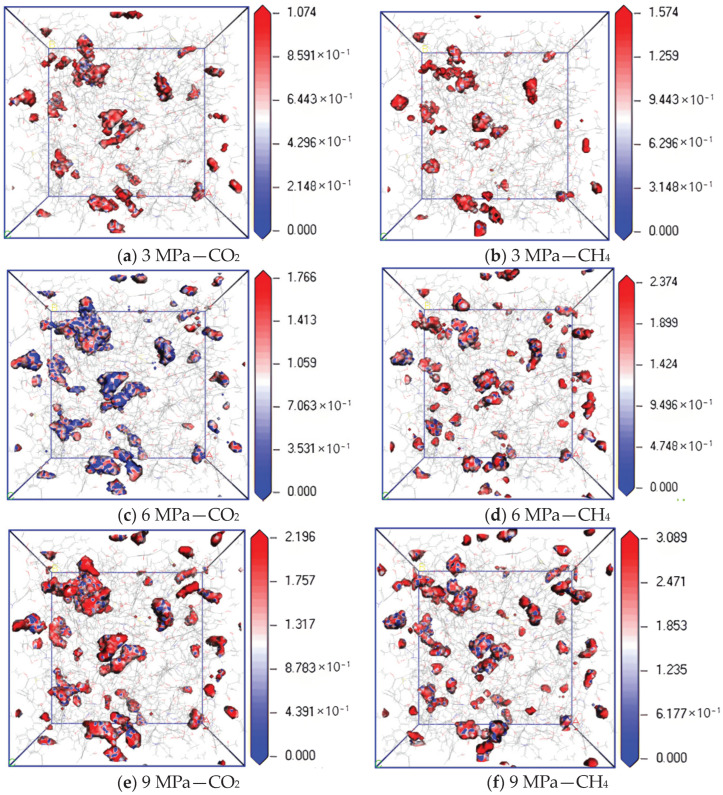
Isopotential value distribution of CO_2_ and CH_4_ at different pressure.

Figure 9 shows the isopotential surface diagram of CO_2_ and CH_4_ changing with adsorption pressure at a temperature of 298.15 K and pressures of 3 MPa, 6 MPa and 9 MPa. It can be found from the simulation results that the equipotential values of CO_2_ and CH_4_ gradually increase with an increase in pressure. The maximum equipotential values of CO_2_ at 3 MPa, 6 MPa and 9 MPa are 1.074, 1.766 and 2.196, respectively, and the maximum equipotential values of CH_4_ are 1.574, 2.374 and 3.089, respectively, indicating that the higher the pressure, the higher the maximum equipotential value. The greater the adsorption capacity of two gases, the stronger the interaction energy between molecules, and the greater the binding degree of gas molecular diffusion. Under the same pressure condition, the equipotential value of CH_4_ is higher than that of CO_2_, indicating that with an increase in pressure, the filling ability of CH_4_ in the micropores on the coal surface is stronger than that of CO_2_, which makes the interaction force between CH_4_ molecules in the micropores stronger, resulting in a greater degree of diffusion obstruction.

### 3.3. Influence of Moisture Content on Diffusion Performance

**Figure 10:**
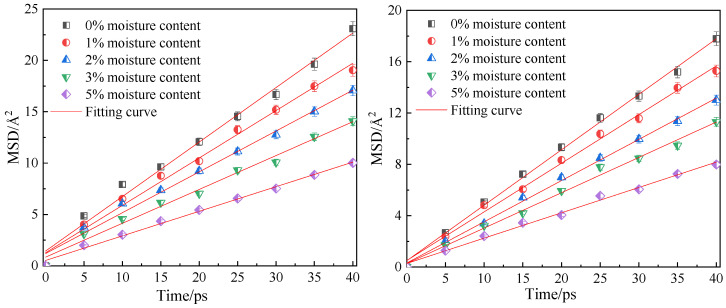
Mean square displacement curve of CO_2_/CH_4_ at different moisture content.

Figure 10 shows the fitting curves of the mean azimuth shift of CO_2_ and CH_4_ when the adsorption temperature is 298.15 K; the adsorption pressure is 5 MPa; and the water content is 0%, 1%, 2%, 3% and 5%. Through linear fitting of the mean azimuth shift curve, the diffusion coefficients of CO_2_ and CH_4_ under different water content conditions were obtained, as shown in Table 3. The simulation results show that with an increase in water content in the coal molecular model, the diffusion coefficients of the two gas molecules CO_2_ and CH_4_ decrease significantly, indicating that water is not conducive to the diffusion of gas in the coal seam. This is because the increase in water content not only occupies the pore space and blocks the diffusion channel of gas in the coal, but also the coal matrix will expand and deform after absorbing water. The effective channel is narrowed and the collision chance between the gas and the hole wall increases, so diffusion is blocked. In addition, water will form ice-like clusters when adsorbed at the adsorption sites on the coal surface [22,24], which can make the micropores clogged.

**Figure 11:**
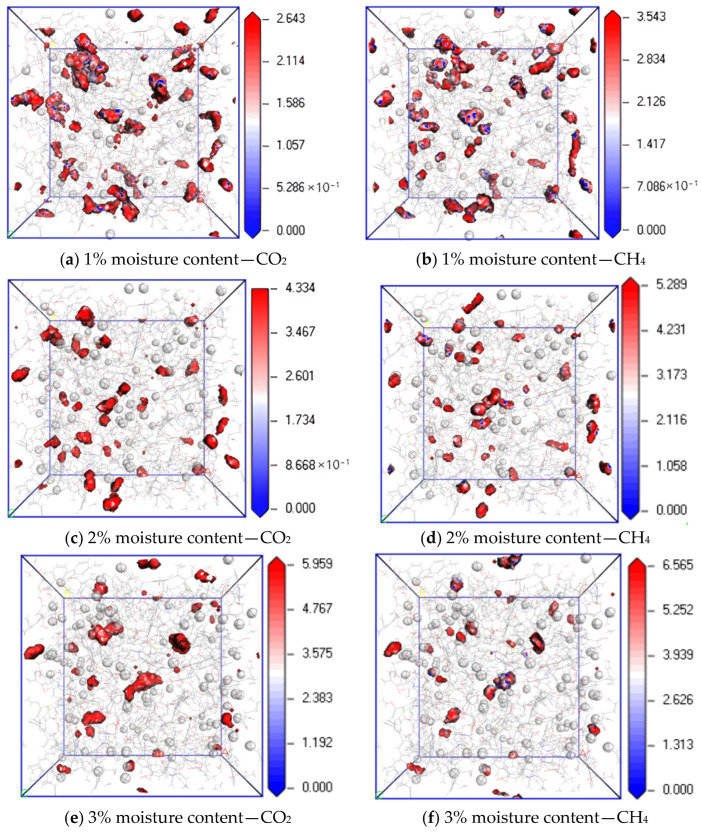
Isopotential value distribution of CO_2_ and CH_4_ at different moisture content.

Figure 11 shows the equipotential surface diagram of CO_2_ and CH_4_ when the temperature is 298.15 K; the pressure is 5 MPa; and the water content is 1%, 2% and 4%. It can be found from the simulation results that when the water content is 1%, 3% and 5%, the maximum equipotential values of CO_2_ are 1.074, 1.766 and 2.196, and the maximum equipotential values of CH_4_ are 1.574, 2.374 and 3.089, respectively. The equipotential values of CO_2_ and CH_4_ gradually increase with an increase in water content. The reason is that an increase in water content in coal makes the H bond between water molecules stronger, which promotes the interaction force between water molecules and the induction force on CO_2_ and CH_4_ molecules. As a result, the potential energy of the system increases with an increase in water content, resulting in the obstruction of gas diffusion. Under the same water condition, the equipotential value of CH_4_ is higher than that of CO_2_ because of the hydration of CH_4_ by H_2_O [25].

## 4. Conclusions

By means of molecular simulation, this paper studies the pore structure characteristics of CO_2_ and CH_4_ gas using a macromolecular structure model of non-cohesive coal in Chicheng Coal Mine, and investigates the influence of different temperatures, pressures and water contents on the diffusion performance of CO_2_ and CH_4_ gas adsorbed by coal and the microscopic mechanism. The main conclusions are as follows:

(1) In the dry-mode macromolecular model, the diffusion coefficients of CO_2_ and CH_4_ gradually increase with an increase in temperature, and a high temperature is conducive to gas diffusion. Under the same conditions, the diffusion coefficient of CH_4_ is lower than that of CO_2_, and the diffusion activation energy of CO_2_ is 4.57 kJ·mol^−1^, while that of CH_4_ is 3.56 kJ·mol^−1^.
(2) In the dry-mode macromolecular model, with an increase in pressure, the diffusion coefficients of CO_2_ and CH_4_ gradually decrease, and the equipotential values of CO_2_ and CH_4_ gradually increase, and a high pressure is not conducive to the diffusion of gas in the coal macromolecular model.
(3) In the water-containing coal macromolecular model, with an increase in water content, the diffusion coefficients of CO_2_ and CH_4_ significantly decrease, and the equipotential values of CO_2_ and CH_4_ gradually increase, and water is not conducive to the diffusion of gas in the coal macromolecular model.

## Figures and Tables

**Table 1:** Diffusivity of CO_2_/CH_4_ at different temperatures of coal samples.

Gas Type	Diffusivity at Different Pressures /×10^−9^ m^2^·s^−1^				
	1 MPa	3 MPa	5 MPa	7 MPa	9 MPa
CO_2_	1.04	0.96	0.89	0.85	0.83
CH_4_	0.77	0.75	0.71	0.67	0.62

**Table 2:** Diffusivity of CO_2_/CH_4_ under different pressures of coal samples.

Gas Type	Diffusivity at Different Pressures /×10^−9^ m^2^·s^−1^				
	1 MPa	3 MPa	5 MPa	7 MPa	9 MPa
CO_2_	1.04	0.96	0.89	0.85	0.83
CH_4_	0.77	0.75	0.71	0.67	0.62

**Table 3:** Diffusivity of CO_2_/CH_4_ under different water content of coal samples.

Gas Type	Diffusion Coefficient under Different Water Content /×10^−9^ m^2^·s^−1^				
	0%	1%	2%	3%	5%
CO_2_	0.89	0.76	0.66	0.55	0.40
CH_4_	0.71	0.63	0.54	0.46	0.33

## Data Availability

The datasets used and/or analyzed during the current study are available from the corresponding author upon reasonable request.
